# Used Daily Dose vs. Defined Daily Dose—Contrasting Two Different Methods to Measure Antibiotic Consumption at the Farm Level

**DOI:** 10.3389/fvets.2019.00116

**Published:** 2019-04-24

**Authors:** Svetlana Kasabova, Maria Hartmann, Nicole Werner, Annemarie Käsbohrer, Lothar Kreienbrock

**Affiliations:** ^1^Department of Biometry, Epidemiology, and Information Processing, WHO Collaborating Centre for Research and Training for Health at the Human-Animal-Environment Interface, University of Veterinary Medicine, Hanover, Germany; ^2^Department for Farm Animals and Veterinary Public Health, Institute of Veterinary Public Health, Vienna, Austria; ^3^Department Biological Safety, Federal Institute for Risk Assessment, Berlin, Germany

**Keywords:** defined daily dose, livestock, treatment frequency, treatment incidence, used daily dose

## Abstract

Tackling the problem of rising antibiotic resistance requires valid and comparable data on the use of antimicrobial drugs in livestock. To date, no harmonized monitoring of antimicrobial usage in animals is available, and there is no system to assess usage data throughout Europe, thus hampering a direct comparison between different European countries. Most of the currently applied monitoring systems are based on sales data. Placement of sales data in relation to the population at risk requires overall assumptions about the weights of the animals treated and the doses applied. Only a few monitoring systems collect data in which the number of treated animals is reported exactly and does not need to be estimated. To evaluate the influence of different calculation methods on the standardizing procedure of antibiotic usage and benchmarking of farms, the treatment frequency for several farms (broiler, suckling piglets, and fattening pigs) was calculated in the following two different ways: first, based on the Used Daily Dose (TF_UDD_), and second, based on the Defined Daily Dose (TF_DDD_). To support this evaluation, consumption data from the Veterinary Consumption of Antibiotics Sentinel (VetCAb-S) project in Germany were used as example data. The results show discrepancies between both outcomes depending on the calculation method applied. In broiler holdings, the median values of TF_DDD_ were 20.89% lower than the median values of TF_UDD._ In suckling piglets and fattening pig holdings, the median values of TF_DDD_ were increased 77.14% and 16.33%, respectively, which may have serious implications for the benchmarking of farms. Furthermore, this finding reflects that the calculation procedure also has an impact on the comparison between populations. Therefore, UDD-based calculations should be preferred to run monitoring systems with a benchmark mission. If, in contrast, the DDD approach is chosen to compare antimicrobial usage between populations, additional considerations should be made to adjust for the addressed discrepancies.

## Introduction

Antibiotic resistance is one of the greatest threats to global health in our century. It can affect anyone, recognizes no borders and leads to higher medical costs, prolonged hospital stays, and increased mortality (1, 2). Although antibiotic resistance evolved long before naturally occurring antibiotics and their derivatives were used to treat human and animal diseases (3), the widespread use of antibiotics in human and veterinary medicine leads to a selective pressure and accelerates this process (4). A central point in establishing an effective strategy to contain antimicrobial resistance in the veterinary sector is to collect and understand data on the consumption of antimicrobials in animals (5). Therefore, standardized indicators of antibiotic usage as well as robust antibiotic monitoring systems are needed. Various indicators are applied to describe antibiotic usage in livestock, the outcomes of which differ and are not always directly comparable (6–8). Currently, no harmonized monitoring system across Europe for antibiotic usage or the assessment of antibiotic usage data exist (9).

Most national reports on antibiotic usage in livestock are currently based on sales data. Sales data are easily available, but they do not provide any information about the treated species, the treatment indication, the number of animals treated or the treatment duration. Evaluating sales data without relation to the potential population at risk and without taking into account the potency and the formulation of drugs has clear limitations (10, 11). There have been several attempts to standardize sales data by taking into account estimates about the treated population to enable comparisons between countries or populations (12, 13).

At the level of the European Union (EU), ESVAC (European Surveillance of Veterinary Antimicrobial Consumption) reports on sales data from 29 EU countries are published annually. In those reports, sales data are harmonized by the animal population by setting the Population Correction Unit (PCU) as a proxy for the animal population at risk in each country. For this calculation, the population at risk of being treated is approximated by the product of the number of individuals at risk of being treated and a standard body weight at treatment (14). The consumption of veterinary antimicrobials is reported in milligrams of active substance per PCU (mg/PCU). Until now, ESVAC has not collected species-specific antimicrobial usage data, and therefore, reports encompass all food-producing animals together, recapped as PCU, precluding the distinction of differences in dosing between species (14).

To enable a more detailed analysis of trends in antimicrobial consumption, ESVAC is striving for the collection of harmonized data on consumption by animal species, as well as a more harmonized calculation method (5). Therefore, “defined daily dose for animals” (DDD_vet_) and “defined course dose for animals” (DCD_vet_) values were established for antimicrobials used in the three major food-producing animal species: pigs, cattle and poultry (broiler) (15). The concept of the Defined Daily Dose for Animals (ADD) was first developed by Jensen et al. (11) and is based on the DDD in humans, where DDD is the assumed average maintenance dose per day for a drug used for its main indication in adult persons at 70 kg body weight. Hence, in humans as well as in the veterinary sector, Defined Daily Doses are nearly always a compromise based on a review of available information, such as recommendations on the Summary of Product Characteristics (SPCs) from different countries (10, 11, 15).

Some European countries, such as The Netherlands and Denmark, have also implemented benchmarking systems at the national level based on the DDD concept (16–19). The Defined Daily Doses used in those benchmarking systems were established at the national level and are not based on the DDD_vet_ published by ESVAC (17).

**Figure 1:**
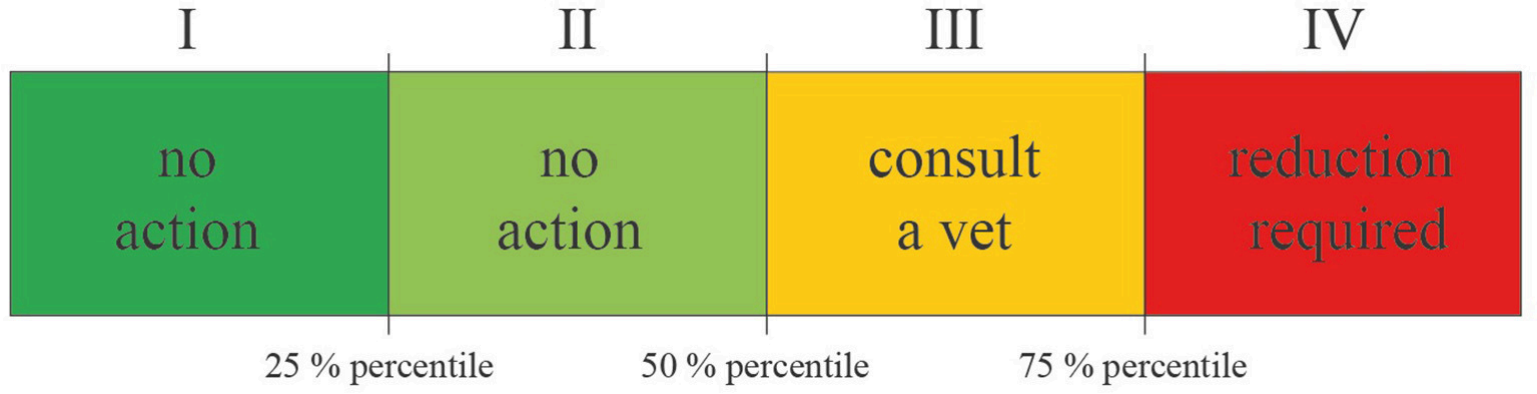
Benchmarking areas of the treatment frequency according to the requirements of the German Medicinal Products Act.

In Germany, in contrast, the Used Daily Dose (nUDD) number per animal directly calculated from the recoded information is applied for benchmarking at the herd level. Other systems utilize a different approach, where the UDD describes the amount of active substance actually administered to the treated animals in mg/kg (20). In contrast to DDD, UDD can only be calculated if the amount of active substance but also the number of treated animals as well as the number of treatment days, is recorded (21). Since the German Medicinal Products Act entered into force in 2014, feedlots for fattening pigs, calves and cattle for meat production and fattening poultry (chicken and turkeys) are required to submit detailed information about each antibiotic treatment and the number of animals kept (22). The treatment frequency (TF) was set up as the benchmarking indicator. Calculation of the TF for all farms separated by species and age group is performed twice a year and officially published by the Federal Office of Consumer Protection and Food Safety. The median and 75% percentile of the TF distribution are defined to be specific benchmark thresholds in this system, as determined for areas with legal regulated actions (see Figure 1) (22).

In our current evaluation of a data subset of the VetCAb-S study collective (23), we aimed to compare two different methods that are used to calculate antibiotic usage at the farm level. We investigated the differences between applying the Used Daily Dose (UDD) and Defined Daily Dose (DDD) to quantify antibiotic consumption and benchmark farms. The aim of the study was to demonstrate the discrepancies between the outcomes of both methods and their impact on the German benchmarking system. We hope that the outcomes of this work can be used as guidance in implementing, evaluating or improving antibiotic usage monitoring systems in livestock at the bottom-up level. Therefore, we calculated the TF based on the nUDD (TF_UDD_), with knowledge of the number of animals treated as well as the treatment duration, following a calculation method very similar to the calculation method established in the German Medicinal Products Act, where only the reference population in the denominator slightly differs. For all these treatment records, we also calculated the TF based on the nDDD (TF_DDD_) by estimating the number of animals treated, considering the amount of active substance delivered to the farmer or applied by the veterinarian and using assumed standard body weights fixed for animals in this production period.

## Materials and Methods

### Study Data

The VetCAb study started in 2008 as a feasibility project to investigate the practicality of implementing an antibiotic monitoring system under the conditions of the German veterinary and agricultural system (24). In 2011, a pilot project was carried out as a cross-sectional study including nearly 3,000 animal holdings across the country (25). Since 2013, the study was continued using a longitudinal approach as VetCAb-Sentinel (VetCAb-S). The study population consists of an open cohort with ongoing participant recruitment, designed to provide a stable study size over time (23). Data collection is related to the mandatory documentation in application and delivery forms (ADF), which is legally required by the German Medicinal Products Act and delivered by farmers or veterinarians to the VetCAb database. These forms include information about the animal species and the number of animals treated, the treatment or delivery date, the treatment indication and application route, the name and amount of the antimicrobial drug applied and, respectively, delivered, and information about the duration of the treatment (26).

From the ongoing VetCAb-S project (23), data on 40 broilers, 135 suckling piglets and 449 fattening pig farms, which participated in the study in 2014, were included in this evaluation. During the time period surveyed, 5% of the broiler farms did not use antibiotics at all. No antibiotic usage was observed in 13.3% of the suckling piglet farms and 14.5% of the fattening pig farms. The treatment frequency was calculated based on the Used Daily Dose (TF_UDD_) following the rules of the German Medicinal Products act and the Defined Daily Dose (TF_DDD_), using DDD_vet_ assigned by ESVAC for pigs and broilers for every active compound and application route (27). Because DDD_vet_ for broilers and pigs were only determined for the oral (broiler) or the oral and parenteral application routes (pigs), respectively, we limited the analyzed dataset exclusively for records of oral and parenteral treatments. Hence, the median of the TF calculated in this particular evaluation may vary from previously published TF where other application routes were also included.

### Treatment Frequency

The treatment frequency is an indicator of the antimicrobial usage in livestock at the farm level, and in Germany it is used as an indicator in the benchmarking system. The TF indicates for how many days, on average, an animal in the observed population is treated within a given time period, e.g., how many single doses were administered to one animal on average within the observation period (21). It describes the number of treatment days per given time period and farm. The treatment frequency meets the classic definition of an incidence of contrasting events in a given population at risk within a defined time period (28).

Within the German benchmarking system, the TF is calculated twice a year according to the following Equation (1) (22):

(1) $$\begin{array}{l} TF=\;\frac{\#\;animals\;treated\;\times \;\#\;treatment\;days\;\times \;\#\;active\;compounds}{\#\;animals\;in\;the\;population\;} \end{array}$$

This calculation method considers the actual number of animals treated, the treatment duration and the number of active compounds in the numerator, and the actual number of animals in the entire farm population in the denominator. The number of active compound depends on the veterinary medicinal product used. Mono-preparations contain only one antimicrobial active ingredient, while combination products contain two or more active substances. Therefore, treatments performed in the same number of animals for the same treatment duration lead to a two-fold higher TF if a combination product, such as sulfonamide/trimethoprim combination treatment, is used [see Equation (1)].

In Equation (1), the amounts used, doses or body weights are only considered indirectly. To include those variables in the calculation, a rearrangement of Equation (1) is needed [see Equation (3) and Equation (4)].

### Used Daily Dose and Treatment Frequency

The Used Daily Dose (UDD) is defined as the actual administered dose per actual kg animal per day. The UDD (mg/kg) can differ between herds and treated animals and must be calculated for every treatment separately (21, 29). In contrast to (1), calculating the UDD in mg/kg requires knowledge concerning the amount of active substance delivered to the farmer, the number and weight of animals treated and the treatment duration (21, 29, 30), as outlined in Equation (2).

(2) $$\begin{array}{l} UDD\left(\frac{mg}{kg}\right)=\frac{amount\;of\;active\;substance\;\left(mg\right)}{\#animals\;treated\times animal\;weight\;\left(kg\right)\times \#treatment\;days} \end{array}$$

Taking into account the amount of antibiotics used, the body weight of the animals treated and the dosage applied, Equation (1) can be rearranged to Equation (3) (30):

(3) $$\begin{array}{l} TF_{UDD}=\frac{amount\;of\;active\;substance\;for\;every\;active\;compound\left(mg\right)}{\#animals\;in\;the\;population\times animal\;weight\left(kg\right)\times UDD\left(\frac{mg}{kg}\right)} \end{array}$$

Hence, the TF_UDD_ calculation method in (3) corresponds to the calculation method for the treatment frequency as shown in (1) and currently applied within the German benchmarking system as laid down by the German Medicinal Products Act (22).

In this paper, the number of livestock places was used in the denominator as a proxy for the animals in the population to calculate the treatment frequency (6, 23, 24, 26). Therefore, the TF_UDD_ as well as the TF_DDD_ calculated within this evaluation indicate how many single doses were administered per livestock place per given time period and farm. Livestock places for piglets were calculated by multiplying the number of livestock places for sows by 10.25, which is the average number of piglets per litter in Germany (23, 31).

### Defined Daily Dose and Treatment Frequency

The Defined Daily Dose (DDD) is the assumed average dose per kg animal per species per day (11, 15). Within monitoring systems, in which antibiotic usage reporting is based on the amount of active substance (16, 18), there is no information about the number of animals treated, and treatment duration or the daily dose actually applied is provided, the treatment frequency can only be estimated by applying standard body weights and Defined Daily Doses, yielding Equation (4).

(4) $$\begin{array}{l} TF_{DDD}=\;\frac{amount\;of\;active\;substance\;for\;every\;active\;compound\;\left(mg\right)}{\#\;animals\;in\;the\;population\;\times \;standard\;animal\;weight\;\left(kg\right)\;\times \;DDDvet\left(\frac{mg}{kg}\right)\;} \end{array}$$

In (4), the number of single doses is estimated by considering the amount of active substance delivered to the farmer standardized by DDD_vet_ and the standard weights of the animals treated. The standard weights considered for the TF_DDD_ calculation correspond to the standard weight proposed by ESVAC (5) and are as follows: suckling piglets (standard weight 4 kg), fattening pigs (standard weight 50 kg) and broilers (standard weight 1 kg). The DDD_vet_ published by ESVAC in April 2016 for pigs and broilers (27) was used for the evaluation. DDD_vet_ is a technical unit and defined to be the assumed average dose per kg animal per species per day (mg/kg), taking into account differences in the dosing, pharmaceutical form and application route used in different species (15). Data on dosing (daily dose and number of days of treatment recommended for the main indication) obtained from the SPCs for antimicrobial veterinary medicinal products were provided for broilers, cattle and pigs by nine EU member states to ESVAC. DDD_vet_ were calculated as the average of all observations of daily doses by species, substance and form (15). As the DDD_vet_ for three long-acting macrolid injectable products, namely, gamithromycin, tildipirosin and tulathromycin, have not yet been published, we set up the DDD based on the Summaries of Product Characteristics of veterinary medicinal products containing these active substances, considering veterinary medicinal products that are only licensed in Germany. Defined Daily Doses were set up as follows: gamithromycin 6 mg/kg, tildipirosin 4 mg/kg and tulathromycin 2.5 mg/kg.

### Benchmarking

To describe the distribution of the TF within the population of farms, the 25% percentile, median and 75% percentile were set as specific benchmark thresholds, resulting in four distribution areas of action (dark and light green: no action, yellow: veterinary consulting useful, red: reduction required, see Figure 1) corresponding to the requirements of § 58 of the German Medicinal Products Act (22). To identify differences in calculation methods, we compared both TF distributions to identify the number of farms in which differences between both outcomes resulted into a shift between action areas within the scope of the German benchmarking system. To demonstrate these differences, cumulative distribution functions were used to show the shift in location and the shape of the distribution. In addition, similarity matrices were employed to describe the number of concordant and discordant results for both measures.

### Estimated Animal Weight at the Time of Treatment

In Germany, the weight of the animals at the time of treatment is not recorded in the ADF forms. Therefore, we calculated the weight of the animals treated for every record following a rearrangement of Equation (2), see Equation (5). In this case, we assumed that the UDD (mg/kg) was the recommended dosage in the SPCs of every veterinary medical product used in the dataset evaluated. For every veterinary medical product used in this evaluation, therefore, we calculated the recommended dosage in mg/kg derived from VETIDATA, a specialized German information platform on questions regarding the usage of medicinal products, toxicology and the legal framework on medicinal products in veterinary medicine (www.vetidata.de).

Information on the amount of active substance, number of animals treated and treatment days is mandatory in ADF forms.

(5) $$\begin{array}{l} \text{animal weight}\left(\text{kg}\right)=\frac{\text{amount of active substance }\left(\text{mg}\right)}{\#\text{ animals treated}\times \text{UDD}(\frac{\text{mg}}{\text{kg}})\;\times \;\#\text{ treatment days }} \end{array}$$

**Figure 2:**
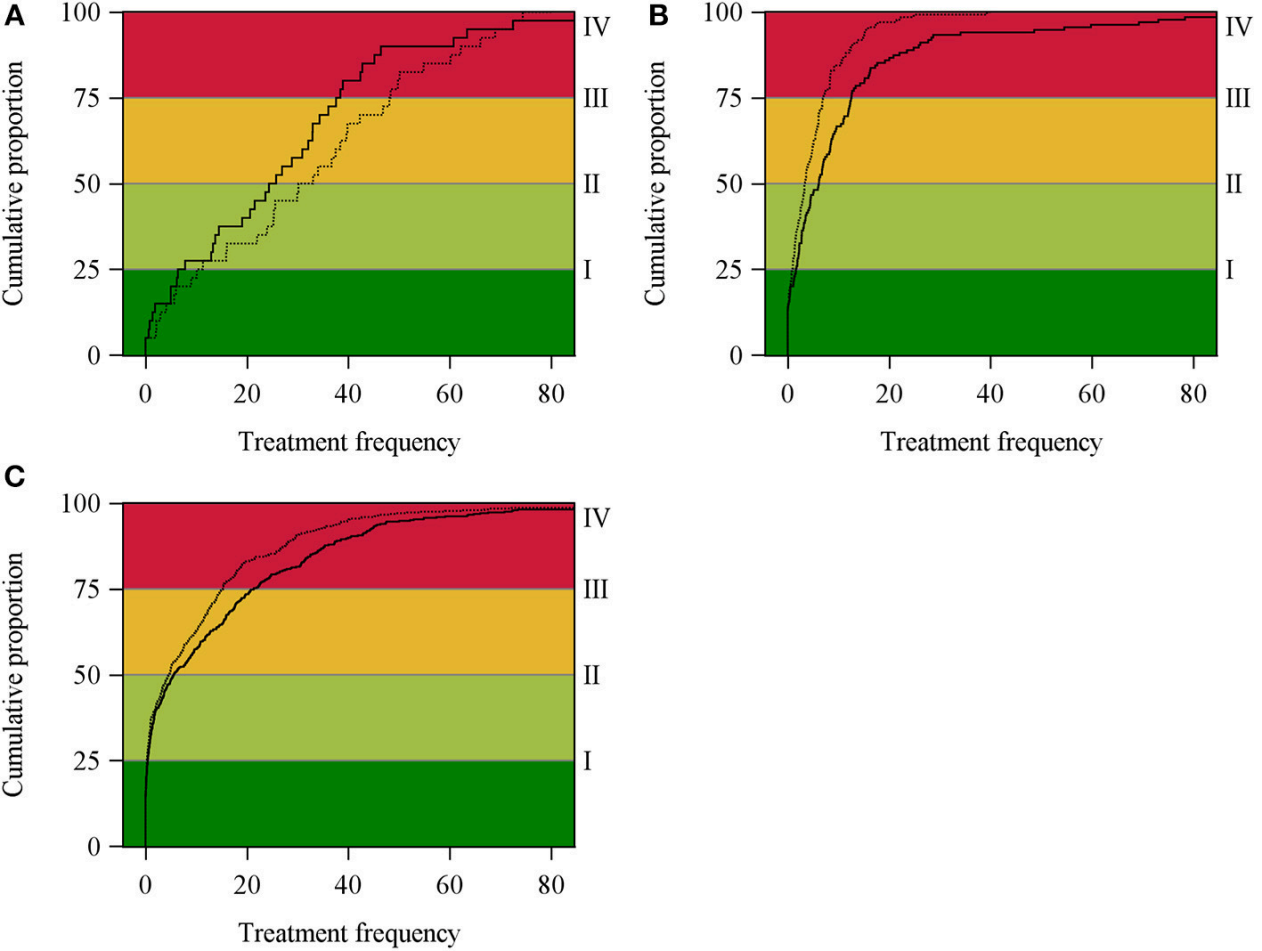
Cumulative distribution function of TF_UDD_ (dashed line) and TF_DDD_ (solid line) in broiler **(A)**, suckling piglets **(B)** and fattening pig **(C)** holdings.

All the statistical evaluations mentioned above were performed with SAS®, version 9.3 TS level 1M2 (SAS Institute Inc., Cary, NC, United States). The graphical representation of the cumulative distribution function of TF_UDD_ and TF_DDD_ shown in Figure 2 was created using the SAS procedure proc univariate.

## Results

### Distribution of Treatment Frequencies Due to the UDD vs. DDD Calculation

Treatment frequencies were calculated for each animal holding following the UDD- and the DDD-concept, respectively (see Table 1). The median of the TF_UDD_ of all suckling piglet holdings was 3.4 with a maximum of 39.3. In the fattening pig holdings, the median of the TF_UDD_ was 4.7. In broiler holdings, the median of TF_UDD_ was 31.6. Based on the DDD, the median of the TF_DDD_ in broiler holdings was 25, and in suckling piglets and fattening pig holdings, it was 6.2 and 5.6, respectively.

**Table 1:** Summary of the treatment frequency for broilers, suckling piglets and fattening pigs based on UDD and DDD.

**Species/age group**	**Number of holdings**	**Minimum**	**5%-Percentile**	**Median**	**Upper quartile**	**95%-Percentile**	**Maximum**
**TF**_**UDD**_							
Broilers	40	0	1.0	31.6	48.2	70.7	74.4
Suckling piglets	135	0	0	3.4	7.1	15.8	39.3
Fattening pigs	449	0	0	4.7	15.2	40.0	409.3
**TF**_**DDD**_							
Broilers	40	0	0.3	25	38	67.9	98.4
Suckling piglets	135	0	0	6.2	12.6	54.5	101.7
Fattening pigs	449	0	0	5.6	21.3	52.1	613.9

Cumulative distribution functions of the TF_UDD_ and TF_DDD_ for broiler, suckling piglets and fattening pig holdings are shown in Figure 2. In broiler holdings (a), the cumulative distribution function of TF_DDD_ generally runs above the cumulative distribution function of TF_UDD_. Within the upper quarter of the distributions, crossing functions are observed indicating substantial differences in the measurements. In contrast, in suckling piglets (b) and fattening pig holdings (c), the cumulative distribution function of TF_UDD_ covers almost the cumulative distribution function of TF_DDD_ in the lower 50% of the data and runs above the function of TF_DDD_ in the upper 50% of the records (see Figures 2A–C).

### Similarity of Benchmarking due to UDD- vs. DDD-Calculation

To demonstrate the shift in both distributions for all species/age groups considered, a similarity matrix for the four areas of action was calculated, showing concordance and discordance in these benchmark areas (see Tables 2–**4**).

**Table 2:** Similarity in benchmarking due to TF_UDD_- and TF_DDD_-distributions for broilers (overall similarity 50%).

**TF_**UDD**_**	**TF**_****DDD****_							
	**I**		**II**		**III**		**IV**	
	**n**	**%**	**n**	**%**	**n**	**%**	**n**	**%**
I	8	80	2	20	0	0	0	0
II	2	20	3	30	1	10	4	40
III	0	0	3	30	5	50	2	20
IV	0	0	2	20	4	40	4	40

*Dark and light green category, no action needed; yellow category, veterinary consulting useful; red category, reduction required*.

In broiler farms, we found the highest discordance among all evaluated production groups. An overall similarity of only 50% indicates a high percentage of farms shifting between categories. Given that neither the first (dark green) nor the second category (light green) are legally restricted, shifts between those categories will not have any consequences for the farmer (Figure 1). This outcome looks different in those cases where there are shifts in or between the third (yellow) and fourth (red) category. In total, 50% of all farms classified to be in the third category (yellow) using the UDD to calculate the TF no longer remained therein using TF_DDD_. Additionally, 20% of those farms shifted into the fourth category (red), and according to the regulations of the German Medicinal Product Act, the development of an action plan would become mandatory for these farms. Finally, 30% shifted into the second category and were no longer subject to any legal regulations (see Table 2).

In 34.1% of all evaluated suckling piglets holdings, there was no match between the categories of TF_UDD_ and TF_DDD_. The highest similarity in benchmarking was found in the first TF category, where only 12.1% of the farms shifted to another category with no legal consequences for the farmer. The lowest similarity was found in the third category, where only 47.1% of the farms remained in the same category if DDD was used (see Table 3).

**Table 3:** Similarity in benchmarking due to TF_UDD_- and TF_DDD_-distributions for suckling piglets (overall similarity 65.9%).

**TF_**UDD**_**	**TF**_****DDD****_							
	**I**		**II**		**III**		**IV**	
	***n***	**%**	***n***	**%**	***n***	**%**	***n***	**%**
I	29	87.9	4	12.1	0	0	0	0
II	4	11.8	20	58.8	9	26.5	1	2.9
III	0	0	9	26.5	16	47.1	9	26.5
IV	0	0	1	2.9	9	26.5	24	70.6

*Dark and light green category, no action needed; yellow category, veterinary consulting useful; red category, reduction required*.

In the group of fattening pigs, we found the highest concordance over all evaluated production groups in the benchmarking of farms (overall similarity 80.4%). We observed 93.8% (dark green), 79.5% (light green), 67% (yellow), and 81.4% (red) similarity in benchmarking for the first, second, third and fourth category, respectively (see Table 4).

**Table 4:** Similarity in benchmarking due to TF_UDD_- and TF_DDD_-distributions for fattening pigs (overall similarity 80.4%).

**TF_**UDD**_**	**TF**_****DDD****_							
	**I**		**II**		**III**		**IV**	
	***n***	**%**	***n***	**%**	***n***	**%**	***n***	**%**
I	105	93.8	7	6.3	0	0	0	0
II	7	6.3	89	79.5	16	14.3	0	0
III	0	0.9	16	14.3	75	67	21	18.8
IV	0	0	0	0	21	18.6	92	81.4

*Dark and light green category, no action needed; yellow category, veterinary consulting useful; red category, reduction required*.

### Distribution of the Estimated Animal Weight at the Time of Treatment

**Figure 3:**
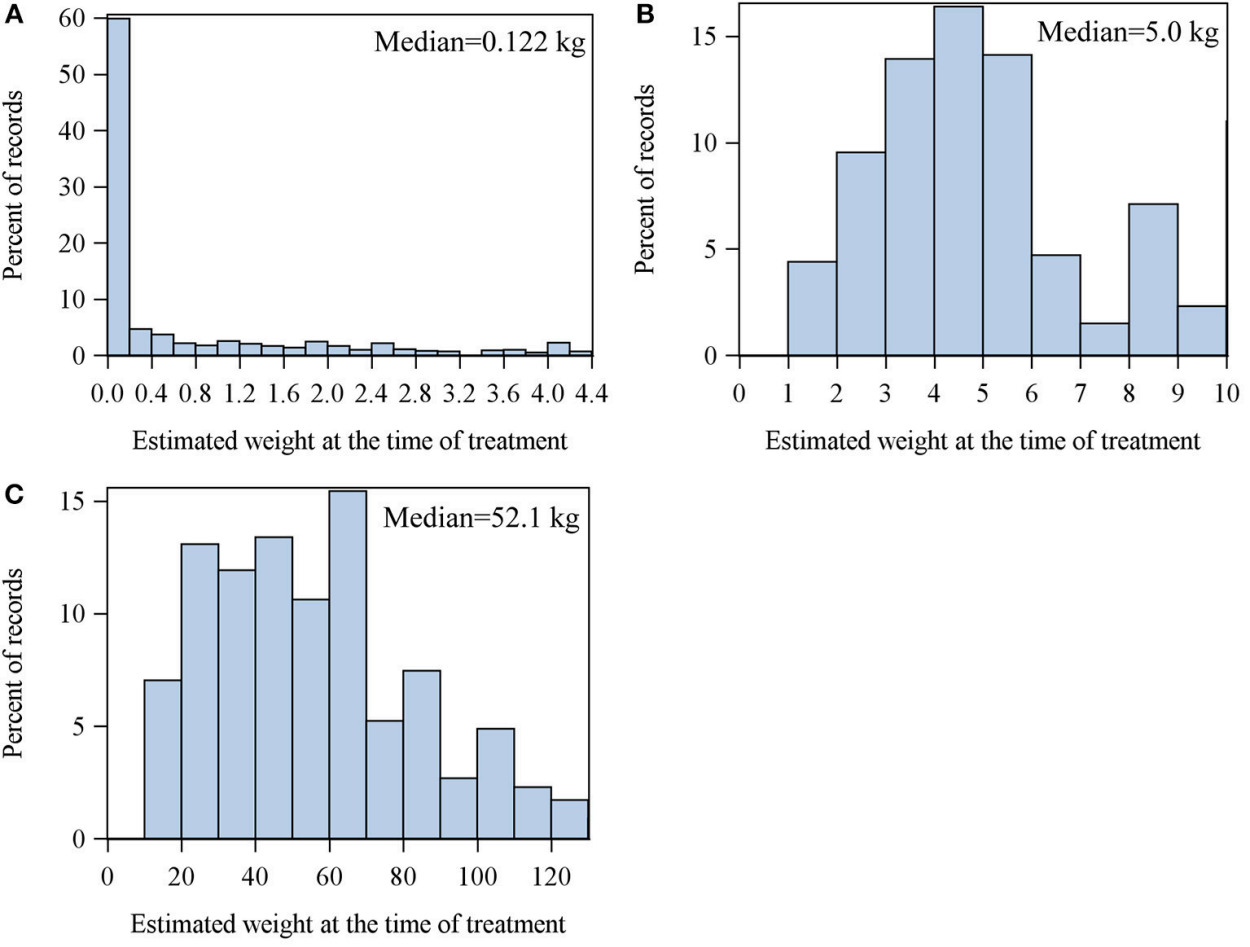
Distribution of the estimated weights of broiler **(A)**, suckling piglets **(B)** and fattening pigs **(C)** at the time of treatment.

Figure 3 shows the distribution of the calculated weight for broilers (A), suckling piglets (B) and fattening pigs (C) based on the ADFs considered in this current evaluation. The median of the estimated weight of the broilers was 0.122 kg, suckling piglets 5 kg and fattening pigs 52.083 kg.

## Discussion

The present work compares two different methods to calculate antibiotic usage in livestock, demonstrating the differences between applying the Used Daily Dose (UDD) and Defined Daily Dose (DDD) and their consequences for individual farmers as well as at the general population level. Both TF calculations are generally in line with the incidence density concept for presenting new events within a given time period (28).

In this evaluation, we used the number of livestock places as a proxy for the animal population at risk (23, 24, 26). The number of livestock places is not exactly equal to the number of animals stabled and maintained during the fattening period, which could vary slightly due to mortality or temporary overcrowding. Those differences between livestock places and the exact number of animas stabled (or present at the farm at any time) can lead to an over- or underestimation of the TF at some point, but we consider that bias to be negligible and compensated by the observation that the number of barn places remains stable over time. In particular, information bias due to under- or misreporting of the number of animals that were stabled or that died during the fattening period can be minimized. Therefore, the number livestock places is more precise and general bias is restricted. This denominator also indirectly considers the observation that there is more than one flock/batch kept per year. The number of treatment days per flock or batch, respectively, could be calculated by dividing the TF calculated per year by the number of flocks/batches per year.

In broiler holdings, the median values of TF_DDD_ were 20.89% lower than the median values of TF_UDD_, while in suckling piglets and fattening pig holdings, the median values were 77.14% and 16.33% higher, respectively. Additionally, the cumulative distribution functions showed similar differences in the shape distributions of TF_UDD_ and TF_DDD_.

Regarding the benchmarking of farms, in 50% of broiler holdings, 34.1% of suckling piglet holdings and 19.6% of fattening pig holdings, the different calculation methods resulted in a shift to another category, potentially associated with varying legal obligations for the farmers.

In a study with a similar approach, Timmerman et al. (29), compared the treatment incidence based on UDD_pig_ (TI_UDDpig_) and ADD_pig_ (TI_ADDpig_) in pigs and found TI_ADDpig_ to be higher than TI_UDDpig._ In this study, ADD_pig_ was estimated based on national dose recommendations from two sources regularly consulted by Belgian veterinarians. The authors considered the discrepancies between TI_ADDpig_ and TI_UDDpig_ to be mainly a consequence of inappropriate dosing, misinterpretations of the leaflet instructions or incorrect evaluations of body weights. Persoons et al. (20), compared TI based on UDD with TI based on DDD in Belgian broiler farms and concluded that, based on UDD, fewer chickens per 1,000 chickens at risk per day were treated than theoretically expected when applying DDD.

Mathematically, differences in both outcomes of the TF are the result of different numbers of single doses used to calculate the TF. By calculating the number of single doses, the amount of active substance (mg) in the nominator always remains the same, regardless of whether the calculation is based on UDD or on DDD. In contrast, the weight of the treated animals and the daily dose considered in the numerator are subject to change, resulting in differences in the number of single doses. Therefore, discrepancies between TF_UDD_ and TF_DDD_ exist for two reasons: primarily, the weight of the treated animals at the time of treatment, considered by calculating TF_UDD_, is not always equal to the standard weight used to calculate TF_DDD_, and second, because UDD is not necessarily equal to DDD.

The weight of animals varies considerably in farming practice. In Germany, broilers are stabled at the age of 1 day (ranging from 1 to 3 days) with a body weight of 40 g (ranging from 38 g to 45 g) and leave for slaughter at the age of 32 to 40 days with an end weight of 1.6 to 2.4 kg. Suckling piglets have a birth weight of 1.5 kg (ranging from 1 to 1.7 kg) and reach 6.9 kg (21-day suckling period) or 8.1 kg (28-day suckling period), respectively, at the time of weaning (ranging from 5.8 kg to 8.8 kg). Fattening pigs are stabled with an average weight of 28 kg (ranging from 25 kg to 30 kg) and leave approximately 115 days later for slaughter with an average end weight of 118 kg (ranging from 110 kg to 120 kg) (32).

Generally, the lower the weight of the treated animals compared with the standard weight, the lower is the treatment frequency of the DDD approach, leading to an underestimation of the TF_DDD_. Conversely, the treatment of animals that are heavier than the standard weight leads to an overestimation of TF_DDD._

Our results showed the TF_DDD_ in broilers was 20.89% lower than TF_UDD_. We consider this underestimation to be mostly due to discrepancies between the standard weight and the real weight of the animals at the time of treatment. The weight of broilers changes by a factor of 40 to 60 during their life span, which carries a high risk of uncertainties in terms of weight estimation. Due to data on treatments in broilers (QS, personnel communication) in Germany, 50% of all treatments take place during the first 7 days of the fattening period, in which the body weight of the animals varies between 40 g and 400 g. In over 70% of the records in our dataset, the weight of the treated broiler was estimated to be <1 kg, likely explaining the underestimation of TF_DDD_ by 20% in relation to TF_UDD_. Therefore, we consider the main reason for the systematic differences in TF calculations to be due to this bias and the differences between UDD and DDD to be of secondary importance in broilers. In suckling piglets and fattening pigs, in contrast, the distribution of the calculated weights of the animals was more symmetric near the standard weights proposed by ESVAC. In contrast to broilers, the weight of suckling piglets changes only by a factor of 5 to 6 on average between birth and weaning. The weight of fattening pigs during a fattening period changes by the factor of 4 on average. Hence, in pigs, systematic errors due to weight variations were lower than in broilers. However, in the estimation of animal weights at time of treatment, we assumed UDD (mg/kg) to be the recommended dosage derived from the SPCs of every veterinary medical product used. Interpreting the distribution of the estimated weight bias due to under- or overdosing needs to be considered.

In addition to the animal weight at treatment, the difference between DDD and UDD must be taken into account. The DDD_vet_ is the assumed average dose per kg animal per species per day and was assigned as an average of the daily doses obtained from Summaries of Product Characteristics (SPCs) for antimicrobial veterinary medicinal products provided for broilers, cattle and pigs by nine EU countries (15). The observations were based on the main indication. DDD_vet_ is a technical unit of measurement that is solely intended for the purposes of drug consumption studies and does not necessarily reflect the daily doses recommended, prescribed or used by the veterinarian's decision.

In contrast, the UDD is the administered dose per kg animal per day determined at the discretion of the veterinarian and dependent on different criteria, such as the veterinary medical product used, clinical picture, pathogenic agents, progression, and spread of the disease, resistance situation, general condition of the patient, etc. The UDD therefore differs between herds, treated animals and veterinarians, and it needs to be calculated for every treatment scenario separately (21). Generally, UDD can also be represented by a statistical distribution within a population under study.

Additionally, systematic differences are observed because the recommended dosage provided in the SPC may vary for the same active substance in and between countries and licensed veterinary medical products. The DDD assigned to be higher than the actually applied UDD leads to an underestimation of the number of single doses and, consequently, a lower TF_DDD_. Conversely, calculations based on a DDD lower than UDD lead to an overestimation of the number of single doses and, therefore, a lower TF_DDD_.

DDD_vet_ for oral and injectable preparations included in the ESVAC document were assigned as an arithmetic mean of all observations for each combination of species, antimicrobial substance and administration route over all products marketed in nine European countries (15). Postma et al. (33), established Defined Daily Dose Animal (DDDA) per active substance and administration route (following the ESVAC approach and using the mean of the recommended dosage for the main indication provided in the SPC) over all veterinary medical products authorized for use in pigs in four European countries (Belgium, France, Germany and Sweden). In their study, (33), found 31 out of 82 unique combinations that showed deviations of >10% from the established consensus DDDA, where most of these products contain tylosin, amoxicillin and doxycycline. Tylosin via the oral application route was the active substance, with the highest difference between the minimum and maximum recommended dosage (1000%).

We compared the recommended dosage based on the SPC for five veterinary medicinal products containing tylosin licensed in Germany for oral medication for pigs as an example. We found the recommended dosage to vary between 4.5 and 25.7 mg/kg body weight depending on the indication, where a main indication could not be identified. The DDD_vet_ for tylosin in oral preparations for pigs is 12 mg/kg. Treating pigs with 4.5 mg/kg as the recommended dosage and estimating the number of treated animals based on a DDD_vet_ of 12 mg/kg in the DDD approach leads to an underestimation of the number of treated animals by a factor of 2.5. Conversely, using 25.7 mg/kg as the recommended dosage leads to an overestimation of the number of animals treated in the DDD approach. We consider such differences between UDD and DDD to have played the major role in discrepancies between TF_UDD_ and TF_DDD_ in our dataset for pigs.

## Conclusion

The results of this evaluation show that the variable used to quantify antibiotic usage has a significant impact on the outcome. It has been demonstrated that the UDD is the most suitable indicator in regard to benchmarking of farms because it represents the real situation on the farm and considers the dosage actually applied as well as the weight of the treated animals. Therefore, we recommend using UDD calculations whenever possible to avoid under- or overestimation of antibiotic usage at the farm level. As a consequence, collection systems for antibiotic usage data need to be expanded with additional information, such as the number of treated animals and the treatment duration. In those cases where using UDD is not an option, e.g., if only sales data are available, one should be aware of the risk of under- or overestimation of the number of animals treated, especially if the treated animals do not reach the standard weight or the national dosages applied substantially differ from the proposed DDD. For broilers, we strongly recommend the standard weight of 1 kg to be adjusted downwards, as we could show that most treated animals had a much lower body weight.

## Ethics Statement

In this study, two different calculation methods of the treatment frequency were evaluated. The data used herein were based on mandatory application and delivery forms, and they were provided voluntarily by farmers and veterinarians after signing individual written consent to the use of the data by the study team only. Our research did not involve any regulated animals, and no scientific procedures were performed on animals of any kind. Thus, formal approval by an ethical committee was not necessary under the provisions of German regulations.

## Author Contributions

SK and LK: conceptualization, formal analysis, investigation, and writing—original draft. SK and MH: data curation. LK: funding acquisition. SK, MH, and LK: methodology. SK: project administration. MH: software. LK: supervision. SK and MH: validation and visualization. SK, NW, AK, and LK: writing—review and editing.

### Conflict of Interest Statement

The authors declare that the research was conducted in the absence of any commercial or financial relationships that could be construed as a potential conflict of interest.

## Abbreviations

ADD: Defined Daily Dose Animal
ADF: Application and Delivery Form
DCD_vet_: Defined Course Dose for animals
DDD: Defined Daily Dose
DDD_vet_: Defined Daily Dose for animals
EMA: European Medicines Agency
ESVAC: European Surveillance of Veterinary Antimicrobial Consumption
EU: European Union
KTBL: Kuratorium für Technik und Bauwesen in der Landwirtschaft e. V.
nDDD: Number of Defined Daily Doses
nUDD: Number of Used Daily Doses
PCU: Population Correction Unit
QS: QS Qualität und Sicherheit GmbH
SPC: Summary of Product Characteristics
TI: Treatment Incidence
TI_UDDpig_: Treatment Incidence based on Used Daily Dose in pigs
TI_ADDpig_: Treatment Incidence based on Animal Daily Dose in pigs
TF: Treatment Frequency
TF_DDD_: Treatment Frequency based on Defined Daily Dose
TF_UDD_: Treatment Frequency based on Used Daily Dose
UDD: Used Daily Dose
UDD_pig_: Used Daily Dose in pigs
VetCAb-S: Veterinary Consumption of Antibiotics Sentinel.
